# Emerging Strategies for Targeting Angiogenesis and the Tumor Microenvironment in Gastrointestinal Malignancies: A Comprehensive Review

**DOI:** 10.3390/ph18081160

**Published:** 2025-08-05

**Authors:** Emily Nghiem, Briana Friedman, Nityanand Srivastava, Andrew Takchi, Mahshid Mohammadi, Dior Dedushi, Winfried Edelmann, Chaoyuan Kuang, Fernand Bteich

**Affiliations:** 1 Department of Surgery, Montefiore Einstein, Bronx, NY 10461, USA; emily.nghiem@einsteinmed.edu; 2 Department of Medicine, Montefiore Einstein, Bronx, NY 10461, USA; 3 Department of Cell Biology, Montefiore Einstein, Bronx, NY 10461, USA; 4 Department of Oncology, Montefiore Einstein, Bronx, NY 10461, USAdior.dedushi@einsteinmed.edu (D.D.); 5 Department of Molecular Pharmacology, Montefiore Einstein, Bronx, NY 10461, USA

**Keywords:** gastrointestinal cancers, angiogenesis, tumor microenvironment, targeted therapy

## Abstract

Gastrointestinal (GI) cancers represent a significant global health burden, with high morbidity and mortality often linked to late-stage detection and metastatic disease. The progression of these malignancies is critically driven by angiogenesis, the formation of new blood vessels, and the surrounding dynamic tumor microenvironment (TME), a complex ecosystem comprising various cell types and non-cellular components. This comprehensive review, based on a systematic search of the PubMed database, synthesizes the existing literature to define the intertwined roles of angiogenesis and the TME in GI tumorigenesis. The TME’s influence creates conditions favorable for tumor growth, invasion, and metastasis, but sometimes induces resistance to current therapies. Available therapeutic strategies for inhibiting angiogenesis involve antibodies and oral tyrosine kinase inhibitors, while immune modulation within the tumor microenvironment is mainly achieved through checkpoint inhibitor antibodies and chemotherapy. Creative emerging strategies encompassing cellular therapies, bispecific antibodies, and new targets such as CD40, DLL4, and Ang2, amongst others, are focused on inhibiting proangiogenic pathways more profoundly, reversing resistance to prior drugs, and modulating the TME to enhance therapeutic efficacy. A deeper understanding of the complex interactions between components of the TME is crucial for addressing the unmet need for novel and effective therapeutic interventions against aggressive GI cancers.

## 1. Introduction

Gastrointestinal (GI) cancers represent a heterogeneous group of malignancies affecting various segments of the GI tract. For classification, these cancers can be broadly categorized into three main groups: upper GI (cancers of the esophagus, stomach, and small intestine), lower GI (cancers of the colon, rectum, and anus), and hepatobiliary (cancers of the pancreas, liver, gallbladder, and bile ducts). The incidence of different types of GI cancers varies globally, with esophageal, gastric, and liver cancers more common in Asia and pancreatic and colorectal cancers more common in Europe and North America [1]. Collectively, this group of diseases contributes significantly to the global cancer burden, accounting for 26% of global cancer incidence and 35% of all cancer-related deaths [1]. Morbidity and mortality rates of GI cancers largely stem from late-stage detection and poor prognosis in the setting of metastatic disease, highlighting the need for further development of treatment modalities [2]. Emphasis should also be placed on prevention and modifiable risk factors such as avoiding diets high in red meat and low in fiber, obesity, sedentary lifestyle, alcohol consumption, and smoking [3].

Though the various types of GI cancers demonstrate differing degrees of reliance on angiogenesis, this process of creating new vasculature is a common driver in the pathogenesis of all these diseases [4]. Once a tumor surpasses 2 mm in diameter, the surrounding tissue can no longer support its growth, creating a hypoxic tumor microenvironment (TME), and subsequently stimulating the formation of new blood vessels to supply oxygen and nutrients [5]. Of the previously mentioned GI cancers, angiogenesis plays the most significant role in cancers of the liver, colon, rectum, and stomach.

Hepatocellular carcinoma (HCC) is a hypervascular tumor, given the unique vascular supply of the liver. It is perfused by both the portal vein and hepatic artery and normally receives approximately 26% of total cardiac output [6]. Vascular endothelial growth factor (VEGF), a key signaling molecule in angiogenesis, has shown high expression in HCC cell lines, tissues, and in the blood circulation of HCC patients—especially those with aggressive disease [7]. Similarly, in the setting of colorectal cancer (CRC), VEGF levels are significantly higher in patients with metastatic disease, with low VEGF expression associated with significantly higher survival rates [8]. Gastric cancer is classified into two major subtypes based on the Lauren classification: intestinal and diffuse types, both differing in their histological features [9]. The intestinal subtype typically metastasizes to the liver via hematologic spread, whereas the diffuse subtype relies on peritoneal dissemination [10]. Their angiogenic phenotypes reflect this discrepancy, with the intestinal subtype showing significantly higher VEGF expression as compared to its counterpart [10].

To the best of our knowledge, this review is the first of its kind focusing on the interplay between angiogenesis and the TME, their role as targets in the treatment of GI cancers, and the drugs that target them. In this review, we define the intertwined roles of angiogenesis and the TME in the tumorigenesis of GI cancers, review the current treatments targeting these two areas, and highlight emerging therapies targeting angiogenesis and the TME.

## 2. Methods

A comprehensive literature search was conducted using the PubMed database to identify relevant articles related to angiogenesis and the TME in GI cancers. The following search terms and Boolean operators were used: (“gastrointestinal cancer” OR “colorectal cancer” OR “gastric cancer” OR “hepatocellular carcinoma” OR “pancreatic cancer”) AND (“angiogenesis” OR “tumor microenvironment”). Studies were included if they discussed key topics (the TME, angiogenesis, treatments for GI cancers targeting the TME and angiogenesis), reported original findings or summarized evidence in systematic reviews, and were published in peer-reviewed journals. Preclinical and clinical manuscripts were included based on primary conclusions and endpoints relevant to the focus of this review.

## 3. The Tumor Microenvironment

**Figure 1:**
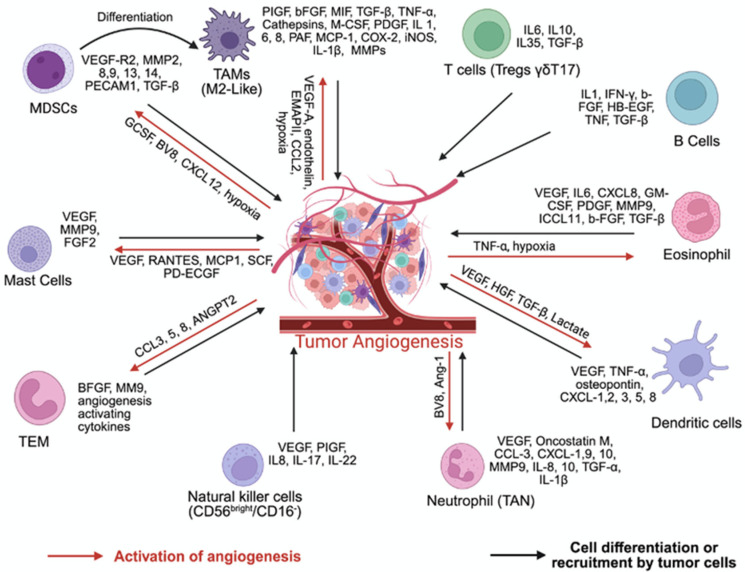
Schematic representation of the TME highlighting the interplay between immune cells and tumor angiogenesis. Mast cells, eosinophils, and dendritic cells contribute to angiogenesis by secretion of pro-angiogenic factors such as VEGF, MMP9, CXCL8, and SPP1. TAMs and TANs release matrix-remodeling enzymes and cytokines that promote endothelial cell activation and vascular remodeling. γδT17 cells secrete IL-17, stimulating VEGF production and angiogenesis. NK cells exhibit a dual role, producing angiogenic factors in low-cytotoxic subsets while cytotoxic subsets may inhibit angiogenesis. Targeted therapies include anti-VEGF agents (e.g., Bevacizumab), MMP inhibitors, and potential IL-17, SPP1, or Bv8 inhibitors. Pharmacologic interventions blocking immune cell recruitment or function (e.g., CSF-1R inhibitors, Bv8 antibodies, MMP14 inhibitors) are approaches to suppress tumor-induced angiogenesis and improve therapeutic outcomes. Created in BioRender. Srivastava, N. (2025) https://BioRender.com/rqnw23g (accessed on 12 June 25).

The TME is composed of various factors, including cellular and non-cellular components that both surround the tumor cells and interact with them. This creates a dynamic environment that continues to evolve throughout the tumor’s progression and results in interactions between tumor cells and the many components that comprise the TME. It encompasses malignant cells and a variety of non-malignant components, including immune cells, stromal cells such as fibroblasts and adipocytes, extracellular matrix (ECM) components, and soluble factors including cytokines, chemokines, and growth factors [11]. The interplay between these constituents critically shapes tumor behavior and therapeutic response. Figure 1 summarizes the components of the TME and the interactions between them.

### 3.1. Immune Cells of the Tumor Microenvironment

Among the immune cells within the TME, CD8+ cytotoxic T lymphocytes are central to antitumor immunity. They mediate direct tumor cell killing through the secretion of perforin, granzymes, and pro-inflammatory cytokines such as interleukin (IL)-2, IL-12, and interferon-gamma (IFN-γ). However, their activity is often attenuated by tumor-expressed ligands, such as PD-L1 and CD80, which engage inhibitory receptors PD-1 and CTLA-4 on T-cells—mechanisms notably targeted by immune checkpoint inhibitors (ICIs). Regulatory T-cells (Tregs) are immunosuppressive T-cells that impede antitumor immunity by inhibiting natural killer (NK) cell cytotoxicity and disrupting interactions between T-cells and antigen presenting cells (APCs). Their infiltration into the TME is frequently associated with poor prognosis in multiple cancer types [11]. B-cells display context-dependent roles based on their phenotype. Within mature tertiary lymphoid structures (TLS), they support increased CD4+/CD8+ T-cell responses and are also associated with reduced Treg populations, leading to an increased immune response, which correlates with improved clinical outcomes [12]. In contrast, memory-like or “exhausted” B-cells expressing CD21/CD27 phenotypes have been linked to immune suppression and tumor progression [13].

Other notable immune cells include NK cells, myeloid-derived suppressor cells (MDSCs), and tumor-associated macrophages (TAMs). NK cells are responsible for antigen-independent cytotoxicity via perforin and granzyme B release. However, they can become functionally impaired in the TME due to hypoxia-induced mitochondrial dysfunction and metabolic stress. In CRC, NK/CD8+ infiltration is associated with better prognosis as opposed to NK/CD4+ infiltration [14,15,16,17]. MDSCs include both monocytic and granulocytic subsets that suppress T-cell activity through the production of reactive oxygen species (ROS), nitrogen species, and arginase. High levels of MDSCs have been associated with increased tumor burden, metastasis, and unfavorable clinical outcomes [18,19,20,21]. TAMs are recruited by hypoxic conditions, necrosis, and tumor-derived cytokines such as CSF1 and IL-6. Most display an M2 phenotype, which promotes immunosuppression, angiogenesis, and metastasis through IL-10, transforming growth factor (TGF)-β, and growth factor secretion. An increased concentration of these TAMs correlates with poor prognosis in CRC [22,23]. While M1 TAMs can prime anti-tumor responses, this phenotype is often underrepresented in the tumor milieu [24].

Tumor-associated neutrophils (TANs) similarly exhibit functional plasticity. The N1 phenotype exerts antitumor activity via recruitment and activation of CD8+ T-cells whereas the N2 phenotype, induced by TGF-β signaling, promotes tumor progression through secretion of growth factors and immunomodulatory cytokines [25,26,27].

### 3.2. Stromal Elements of the Tumor Microenvironment

The stroma comprises numerous cell types, some of which contribute to tumor progression, such as cancer-associated fibroblasts (CAFs), adipocytes, and mesenchymal stem cells (MSCs). CAFs promote tumorigenesis and immune evasion through ECM remodeling, secretion of growth factors, and immunosuppressive activities. They also enhance resistance to chemotherapy and immunotherapies by modulating drug uptake and shielding tumor cells [28]. Adipocyte phenotype and function can be altered in the setting of obesity, subsequently increasing inflammation and hypoxia in the TME. MSCs can be reprogrammed by gastric cancer to promote tumor growth and immunosuppression, as well as enhance angiogenesis via IL-6 secretion [29,30].

### 3.3. The Tumor Microenvironment and Angiogenesis

The TME contributes to the pathological angiogenic process, which creates favorable conditions for tumor growth and survival, supporting their expansion, spread, and invasion, followed by metastasis formation [31,32,33]. During tumor development, inflammatory signals recruit macrophages to the tumor stroma, including chemokines (e.g., CCL2, CCL5, CXCL12), cytokines (e.g., VEGF, CSF1), and complement factors [34]. Evidence supports that chemokines such as IL-8 can promote tumor angiogenesis in non-small-cell lung cancer, colorectal cancer, and glioma cells while others block it (e.g., CXCL10) [35,36]. The balance between these signals determines whether tumors can grow and spread by forming new blood or lymphatic vessels. In addition, these signals convert circulating monocytes, monocytic MDSCs, or resident macrophages into TAMs, which serve as key links between chronic inflammation and cancer progression, promoting tumor invasion, angiogenesis, lymphangiogenesis, and metastasis.

#### 3.3.1. Tumor-Associated Macrophages

In primary tumors, TAMs release proangiogenic factors, such as VEGF-A, EGF, FGF-2, CXCL8, and TNF-α, to stimulate endothelial cell activation and new vessel formation. TAMs also contribute to lymphatic vessel growth via VEGF-C and VEGF-D and facilitate ECMremodeling through matrix mellanoproteases (MMPs) such as MMP2, MMP9, uPA, and cathepsins, aiding in tumor invasion and spread [34]. In an inflammation-driven colon cancer model, Suarez-Lopez et al. identified TAM-derived CXCL12 as a major proangiogenic factor [24]. CSF-1 upregulates Tie2 in TAMs in gastric cancer, linking them to vessel formation and tumor cell intravasation. Tie2^+^ TAMs align along vasculature and promote invasion via a CSF-1–EGF feedback loop. They also support pre-metastatic niche formation through LOX-induced collagen crosslinking and MMP-2 release, enhancingECM. Furthermore, TAM-derived SPARC, cathepsins, and TGF-β collectively facilitate epithelial–mesenchymal transition (EMT), angiogenesis, and immune evasion, subsequently driving tumor progression [37,38,39].

#### 3.3.2. Tumor-Associated Neutrophils

TANs are increasingly recognized as another important mediator of tumor angiogenesis and metastasis in mice and humans [40,41]. Their proangiogenic function is primarily attributed to the secretion of factors such as IL-1β, VEGF, FGF-2, TGF-α, HGF, Ang1, and chemokines, including CXCL1, CXCL8, CXCL9, CXCL10, CCL3, and CCL4. TANs also release MMP9, contributing to remodeling and forming ECM vessels [42]. Neutrophil production is driven by CSF3 (G-CSF) signaling through CSF3R, activating STAT3, which induces Bv8 upregulation, a key factor in promoting myeloid-driven angiogenesis [43]. STAT3 signaling regulates the angiogenic activity of TANs via induction of VEGF-A, FGF-2, and MMP9. In early carcinogenesis models such as RIP1–Tag2 mice, MMP9+ neutrophils initiate the angiogenic switch. Depletion of neutrophils in these models reduces VEGF/VEGFR signaling and delays angiogenesis [44]. Notably, TANs lack tissue inhibitor TIMP1, making their MMP9 more effective in driving angiogenesis. In contrast, under certain conditions such as in PPARα-deficient mice, TANs may exhibit antiangiogenic behavior by releasing thrombospondin-1 [45]. Neutrophils also express secreted phospholipase A2, enhancing VEGF-A, Ang1, and CXCL8 production, but also inducing the antiangiogenic VEGF-A165b isoform, suggesting a balance between pro- and antiangiogenic factors [46].

TANs also support metastasis by forming neutrophil extracellular traps (NETs) and establishing the pre-metastatic niche. Vincent et al. identified that elevated lysyl oxidase-like 4 (LOXL4) expression in neutrophils contributes to resistance against antiangiogenic therapy in CRC, highlighting a novel role for LOXL4 in neutrophil-driven angiogenesis [47]. Additionally, Bv8 (prokineticin 1/2) levels are markedly increased in CRC patients and are highly expressed in TANs in mouse models [43]. Bui et al. identified TANs as a primary source of MMP14 and osteopontin (OPN) in CRC, showing that each factor distinctly contributes to tumor angiogenesis through VEGF-independent mechanisms [42].

Itatani Y. et al. demonstrated that anti-VEGF therapy in VEGF-resistant CRC mouse models increases plasma G-CSF levels, which promotes neutrophil infiltration and enhances angiogenesis via upregulation of Bv8 in TANs. Notably, targeting neutrophil-derived Bv8 improved the effectiveness of anti-VEGF antibody therapy, underscoring its potential as a therapeutic target [43]. Recent findings also indicate that TANs and TAMs contribute to resistance against antiangiogenic therapy. Tumors activate PI3K signaling in CD11b+ myeloid cells (both neutrophils and monocytes); blocking one subset triggers compensatory activation of the other. Dual inhibition of PI3K in all CD11b+ cells leads to sustained angiogenesis suppression [48].

#### 3.3.3. Mast Cells

Mast cells (MCs) promote angiogenesis by releasing factors such as VEGF-A, FGF-2, TNFs, and CXCL8, proteases such as MMP9 and chymase, and tryptases, which activate pro-MMPs. Studies in MC-deficient tumor-bearing mice have shown reduced angiogenesis and metastasis, highlighting their pro-tumorigenic role [49,50]. In CRC, tryptase-positive MCs correlate with increased angiogenesis [51]. Tryptase is considered a central mediator of MC-driven tumor angiogenesis, cell proliferation, and invasion, making it a potential therapeutic target. Initially developed for allergic conditions, tryptase inhibitors show promise as antiangiogenic and anti-tumor agents and could be repurposed, potentially in combination with immunotherapy. Additionally, Nagaoka et al. showed that IL-17-driven recruitment of TANs in a progressive gastric cancer model significantly enhances tumor angiogenesis and contributes to tumor progression [52]. Supporting this, Li et al. identified IL-17^+^ neutrophils as a predominant source of MMP-9 at the invasive margins of gastric tumors [53]. These findings highlight a critical proangiogenic role of TANs in gastric cancer, particularly through IL-17-mediated signaling and MMP-9 production. These collectively foster a microenvironment conducive to tumor growth and vascularization.

#### 3.3.4. γδT17 Cells

γδT17 cells are a subset of lymphocytes that secrete IL-17 and contribute to tumor angiogenesis. High IL-17 expression has been linked to increased microvessel density and VEGF production in CRC, serving as an independent predictor of poor overall survival (OS) [53,54,55,56]. In gallbladder cancer (GBC), γδT17 cells are enriched in both blood and tumor tissues. Their presence, alongside TH17 and Treg cells, is associated with worse prognosis [57]. IL-17 stimulation of GBC cell lines (e.g., OCUG-1) leads to VEGF upregulation, confirming a functional proangiogenic role of γδT17 cells [58]. Angiogenesis array analysis of γδT17-derived supernatants revealed IL-17-driven increases in angiogenic factors such as VEGF, angiogenin, uPA, MMP9, CCL2, CXCL16, CSF2, and tissue factor, alongside elevated antiangiogenic molecules including thrombospondin-1, TIMP1, serpine-1, and PF4 [48,59].

#### 3.3.5. Innate Lymphoid Cells

Innate lymphoid cells (ILCs) are classified into ILC1, ILC2, and ILC3 subsets based on cytokine profiles and transcription factor dependencies. ILC3s, which express RORγt and produce IL-17 and IL-22, are implicated in tumor-promoting inflammation, particularly in the colon. Their pro-tumorigenic functions are largely driven by IL-23-mediated activation, leading to chronic inflammation, tumor progression, and angiogenesis. Like γδT17 cells, ILC3s contribute to tumor growth by releasing IL-17, promoting VEGF expression and vascular remodeling [60,61]. Both γδT17 cells and ILC3s support the recruitment of immunosuppressive cells, including MDSCs, Tregs, and M2-like macrophages, thereby shaping a proangiogenic and immunosuppressive TME. Moreover, ILC3-derived IL-22 further enhances tumor development in bacteria-driven CRC models, underscoring functional parallels with γδT17 cells in inflammation-driven tumorigenesis [60,61,62].

#### 3.3.6. Intracellular Modulators

Tumor angiogenesis is regulated not only by extracellular factors such as cytokines and growth factors but also by intracellular modulators, including non-coding RNAs. These RNAs can be delivered into tumor cells via exosomal and non-exosomal pathways. Long non-coding RNAs (lncRNAs) have emerged as key regulators of angiogenesis. In CRC, lncRNA MALAT1 enhances VEGF-A expression by acting as a competing endogenous RNA (ceRNA) for miR-126-5p and miR-3064-5p. MALAT1 promotes angiogenesis and vasculogenic mimicry in gastric cancer through the VE-cadherin/β-catenin axis. Similarly, lncRNA ZFAS1 facilitates angiogenesis via the miR-150-5p/VEGF-A signaling pathway [63].

## 4. Angiogenesis

One final cell type of the TME not mentioned in the previous section is vascular cells. Blood vessels are lined by a single-cell layer comprising endothelial cells (ECs) [64]. In small blood vessels, ECs are encased by pericytes, mesenchymal cells embedded in the basement membrane that stabilize ECs and limit vascular permeability. In the setting of tumorigenesis, pro-angiogenic signals can lead to the disruption of pericyte coverage and facilitate metastasis. These same signals can also stimulate ECs, resulting in aberrant growth and branching of blood vessels [31]. This section discusses the signaling pathways that affect the vascular cells of the TME.

**Figure 2:**
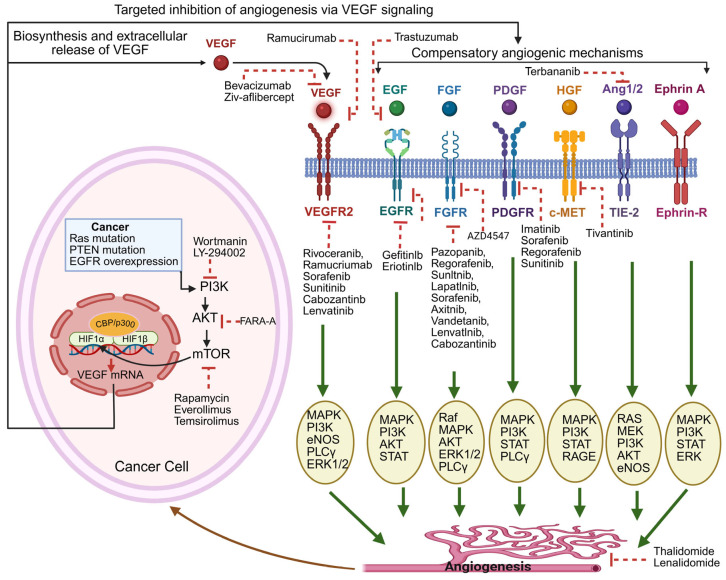
Angiogenic signaling pathways and molecular targets of anti-angiogenic therapies discussed in this review. This schematic depicts the key signaling interactions between cancer cells and ECs that drive tumor angiogenesis, alongside the molecular targets of various anti-angiogenic agents. Under hypoxic conditions, tumor cells stabilize and upregulate HIF-1α and HIF-1β, which, in cooperation with transcriptional co-activators such as CREB-binding protein (CBP), promote the transcription of VEGF. Secreted VEGF binds to VEGF receptors (VEGFRs) on adjacent ECs, initiating downstream signaling cascades that promote EC activation, proliferation, and neovascularization. Pharmacological agents such as rapamycin, everolimus, and other mTOR inhibitors disrupt mTOR-mediated signaling, which is critical for cell growth and angiogenic responses. While VEGF-targeted therapies can effectively inhibit primary angiogenic pathways, tumors often activate compensatory signaling through alternative pro-angiogenic ligands such as epidermal growth factor (EGF), FGF, PDGF, and hepatocyte growth factor (HGF). The diagram also highlights the mechanisms of action of various tyrosine kinase inhibitors (TKIs), which block multiple receptor-mediated angiogenic signals. Ang1, Ang2, and Ephrin-A ligands also contribute to vascular remodeling by binding to their respective Tie and Eph receptors, representing additional nodes of angiogenic regulation and potential therapeutic intervention. Created in BioRender. Srivastava, N. (2025) https://BioRender.com/1invh7u (accessed on 25 July 2025).

As tumors progress, they often stimulate the growth of new blood vessels to sustain their progression [38]. When tumor cell growth outpaces the capacity of existing vasculature, regions of low oxygen develop, and in response, tumors begin producing factors that promote blood vessel formation in a disorganized and excessive manner [65]. This response is largely controlled by a group of proteins called hypoxia-inducible factors (HIFs), which become stabilized under low-oxygen conditions and activate genes involved in blood vessel growth such as VEGF, fibroblast growth factor (FGF), and platelet-derived growth factor (PDGF) [66,67]. High levels of HIF-1α are frequently seen in GI malignancies and are linked to worse clinical outcomes as well as poor response to therapy [68,69]. Tumors can access the blood supply in two main ways: either by inducing the growth of new vessels or by utilizing nearby pre-existing vessels, a process known as vessel co-option [70]. The point at which tumors begin actively promoting vessel growth is often referred to as the “angiogenic switch” and represents a key step in tumor development [71]. Multiple growth factors and their receptors are involved in the formation of new blood vessels within tumors. These include VEGF, FGF-2, PDGF, angiopoietins, ephrins, apelin, and chemokines. They often act together, each contributing at different stages of vessel development [38]. Figure 2 summarizes the signaling pathways detailed in this section.

### 4.1. VEGF Signaling

The VEGF family includes VEGF-A (commonly called “VEGF”), VEGF-B, VEGF-C, VEGF-D, and placental growth factor (PlGF). VEGF-A, produced by tumor and surrounding stromal cells, triggers the growth and survival of ECs, leading to the formation of abnormal, often leaky blood vessels [72]. VEGF is commonly overexpressed in human cancers and is associated with increased invasiveness, higher vessel density, greater metastatic potential, and worse prognosis [73,74].

### 4.2. FGF Pathway

Fibroblast growth factors (FGFs), through their interaction with FGFRs, influence both cancer and stromal cells, promoting cell growth, resistance to death signals, movement, and new blood vessel formation [75]. FGF-2 (also known as bFGF) is the best-characterized member of this group in both normal and cancer settings [76]. Importantly, FGF signaling can contribute to resistance against VEGF-targeted therapies, offering tumors an alternative route to maintain angiogenesis when VEGF is blocked [77].

### 4.3. PDGF Signaling

PDGF and its receptors support tumor growth through both direct effects on cancer cells and indirect effects on surrounding stromal cells. PDGF contributes to vessel formation by recruiting and activating supporting cells in the TME [78].

### 4.4. Angiopoietin/Tie2 Axis

Angiopoietins, especially ANGPT-2, play a role in shaping the tumor vasculature. ANGPT-2 can destabilize existing vessels, detach pericytes, and encourage new vessel sprouting [79]. Blocking both ANGPT-2 and VEGFR2 has shown benefits in preclinical models. Wu et al. assessed the efficacy of Ang2 and VEGF blockade in various murine cancer models (colorectal, breast, liver, and renal) in the setting of postsurgical metastatic disease. This study found that, though not effective against primary tumors, combinations of Ang2 and VEGF blockade were able to slow the progression of metastases, with different combinations producing varying levels of efficacy across the different cancer types [80]. These promising preclinical results warrant further investigation to better elucidate the best combinations of Ang2 blockade for each cancer type.

### 4.5. Eph/Ephrin System

Eph receptors and their ephrin ligands are often upregulated in cancers such as colorectal, breast, liver, and brain tumors [81,82]. While overexpression of these molecules can promote tumor growth and spread, their loss can also contribute to cancer progression, as shown with reduced EphA1 levels in CRC being linked to increased metastasis [83].

### 4.6. Apelin/APJ Pathway

Apelin, a small peptide, binds to the APJ receptor and is upregulated in several cancers, including colorectal cancer [84,85]. Its expression increases under hypoxic conditions [86], and it contributes to tumor growth by supporting the development of new micro-vessels [87,88].

### 4.7. Chemokine-Driven Angiogenesis

Chemokines are signaling proteins that work through G-protein-coupled receptors to guide immune cells. Some chemokines also promote angiogenesis, particularly through the CXCR2 receptor [89,90]. Another receptor, CXCR4, supports new vessel formation when activated by its ligand CXCL12 [81,91].

### 4.8. TGF-β Pathway

TGF-β has a dual role in cancer, but in the context of angiogenesis, it tends to promote blood vessel growth by increasing VEGF and other pro-angiogenic signals [92,93].

## 5. Current Therapies—Targeting the Tumor Microenvironment

Supplementary Materials Table S1 lists the molecular formula and chemical structure, when applicable, of drugs mentioned in Section 5, Section 6, Section 7 and Section 8.

### 5.1. Chemotherapy

A cornerstone of GI cancer treatment is conventional cytotoxic chemotherapy agents such as 5-fluorouracil (5-FU), oxaliplatin, and irinotecan. They not only directly affect tumor cells, but they can also significantly alter the TME via immune cell population modulation and via cytokine networks. 5-FU causes DNA damage and also activates the STING pathway, one that is intrinsic to cancer cells and results in a pro-inflammatory immune response. However, it can also lead to the promotion of MDSC expansion and Th-17-skewed cytokine profiles, which ultimately leads to the suppression of effective anti-tumor immunity [94,95]. Another limitation of this therapy is non-coding RNA-mediated resistance due to modulation of the TME and tumor cell behavior [95]. Oxaliplatin is known to induce immunogenic cell death, facilitating DC uptake and the activation of T-cells. It is also involved in TME remodeling, which, in pre-clinical models, has been shown to increase the efficacy of checkpoint blockade therapies [96,97]. A study by Gou et al. demonstrated that, in addition to its cytotoxic effects, intraperitoneal administration of oxaliplatin reduced the number of TAMs and MDSCs in a murine colon cancer model, decreasing immunosuppression and allowing for an enhanced antitumor immune response [96]. Irinotecan has been shown to be effective in CRC, but there is known resistance due to a variety of factors affecting the TME, including intercellular vesicle-mediated transmission of miRNAs [98]. It must be noted that while chemotherapy is the mainstay of current treatment of GI malignancies, there are many adverse effects that can often be treatment-limiting or can persist even after treatment completion, leading to a dramatic negative impact on a patient’s quality of life [99].

### 5.2. Targeted Therapies

In addition to chemotherapy, multiple approved targeted therapies utilize the driving molecular features of tumors to control them and have immunomodulatory effects on the TME. One such treatment is trastuzumab, a monoclonal antibody that has been used to target HER2-expressing gastric cancer. Its activity is bolstered by a robust NK cell presence within the TME, as this allows for increased antibody-mediated cytotoxicity. However, there has been noted resistance to this drug in gastric cancer cells due to glutamine metabolism and increased M2-phenotype macrophages [100,101]. Other agents affect the crossover between angiogenesis and the TME, such as ramucirumab and bevacizumab, which target VEGFR2 and VEGF, respectively. Ramucirumab alters the TME through the reduction in angiogenesis. It also acts on the TME by increasing CD8+ T-cell numbers and limiting various immunosuppressive cells, leading to improved outcomes in both gastric and gastroesophageal junction (GEJ) cancers [102,103]. Cetuximab is a monoclonal antibody that not only blocks EGFR-mediated proliferative signals in RAS wild-type CRC tumors but also targets the TME. It acts on TAMs and encourages their reprogramming into the pro-inflammatory M1 phenotype, which leads to greater anti-tumor functioning [104,105]. These tailored therapies are useful because of their ability to directly target specific aspects of tumors, but they also face resistance. For example, gastrointestinal stromal tumors (GISTs) initially respond to tyrosine kinase inhibitors (TKIs) such as imatinib, but ultimately, tumors develop secondary mutations that result in resistance and limitations in drug effectivity.

### 5.3. Immunotherapy

Immunotherapies are among the approved cancer treatments known for their impact on the TME. These ICIs have potent effects across multiple tumor types. Pembrolizumab, a PD-1 inhibitor, is effective in a small subset of microsatellite-high (MSI-H) CRCs and in a large proportion of gastric cancers. It modulates the TME by affecting the populations and activities of cell types present within it. Pembrolizumab causes a reduction in Treg cells, improves CD8+ T-cell activity, and encourages long-lasting immune memory. Its anti-tumor effectivity is strongly tied to the presence of a pre-inflamed TME [106]. Another notable medication is nivolumab, a therapy that is approved for use in advanced gastric and GEJ adenocarcinomas. It facilitates the infiltration and subsequent activation of various cytotoxic lymphocytes into the TME [107].

Similarly to the other approved medications, ICIs, while effective and overall safe, still face limitations. Firstly, there is limited applicability of these medications, as most GI cancer patients do not exhibit the specific targets of these medications. For example, most GI tumors are microsatellite-stable (MSS) or have a cold TME, and thus, a medication such as pembrolizumab would not be as effective [108]. Additionally, ICIs can lead to serious immune-related adverse effects, resulting in the need for immunosuppressive treatment or even premature discontinuation of the cancer treatment. A retrospective study of patients with CRC receiving ICIs found that 3.8% of study participants had immune-mediated colitis, with a large percentage ultimately needing corticosteroids, with some requiring even stronger immunosuppressant medications [83].

Overall, while there are several treatment options available that target the TME quite successfully, the limitations surrounding these treatments necessitate continued research and development of new or improved treatment modalities.

## 6. Approved Anti-Angiogenic Drugs for Gastrointestinal Malignancies

Inhibiting angiogenesis has become a key strategy in cancer therapy. Several anti-angiogenic drugs have received FDA approval and are currently used in clinical practice. These drugs target different components of the angiogenesis signaling pathway, either by blocking the ligands, their receptors, or related downstream signals. Below is a summary of the main agents used, their mechanisms of action, and the specific GI cancers for which they are approved or under investigation. Table 1 and Table 2 list the various drugs and drug combinations reviewed in this section.

### 6.1. Anti-Angiogenic Monoclonal Antibodies

#### 6.1.1. Bevacizumab

Bevacizumab is a monoclonal antibody that binds to plasma VEGF-A, preventing it from activating its receptor [109].

It is approved for first and second-line treatment of metastatic colorectal cancer (mCRC) in combination with fluorouracil-based chemotherapy. The Eastern Cooperative Oncology Group Study enrolled 829 mCRC patients treated previously with fluoropyrimidine and irinotecan and assigned them to one of three groups: FOLFOX4 with bevacizumab, FOLFOX4 alone, or bevacizumab alone. They found that the combination group had a median survival duration of 12.9 months as compared to 10.8 and 10.2 months for the FOLFOX4 group and bevacizumab groups, respectively (HR for death = 0.75; *p* = 0.0011). Median progression-free survival (PFS) was 7.3 months in the combination group as compared to 4.7 months and 2.7 months in the FOLFOX4 group and bevacizumab groups, respectively (HR for progression = 0.61; *p* < 0.0001). Overall response rates (ORRs) were 22.7% compared to 8.6% and 3.3% respectively *(p* < 0.0001 for combination vs. FOLFOX4 comparison) [110]. Bevacizumab can indirectly improve the efficacy of FOLFOX4 via its effects on the TME [111]. The hypoxic conditions exacerbated by tumor angiogenesis and lactic acid production from the Warburg effect can lead to decreased uptake of chemotherapy agents due to drug protonation and neutralization [112]. Thus, the anti-angiogenic mechanism of bevacizumab, which mitigates hypoxia in the TME, can synergistically increase the delivery and efficacy of FOLFOX4.

Bevacizumab is also approved in combination with atezolizumab, an anti-PD-L1 agent, as a first-line option for unresectable HCC [113]. IMbrave150 was a global, open-label phase III clinical trial involving 501 patients at 111 sites in 17 countries with unresectable HCC and without prior systemic treatment. Patients were randomly assigned in a 2:1 ratio to either a bevacizumab with atezolizumab group or an oral sorafenib group. OS at 12 months was 67.2% and 54.6% for the combination and sorafenib groups, respectively (HR for death for combination vs. sorafenib = 0.58; 95% CI = 0.42 to 0.79; *p* < 0.001)). Median PFS was 6.8 and 4.3 months, respectively (HR for disease progression or death = 0.59; 95% CI, 0.47 to 0.76; *p* < 0.001) [113]. VEGF creates an immunosuppressed TME by increasing immunosuppressive cells such as MDSCs, M2-TAMs, and Tregs. By targeting VEGF binding, bevacizumab counteracts these effects to create an immunoreactive TME that can synergistically improve the efficacy of immunotherapy agents such as atezolizumab [114].

Additionally, a new combination of bevacizumab and trifluridine/tipiracil (FTD-TPI), a nucleoside analogue, has been approved after showing improved outcomes in mCRC patients who had previously received treatment, including patients previously treated with bevacizumab alone. The SUNLIGHT phase III trial compared bevacizumab and FTD-TPI with FTD-TPI alone. Median OS was 10.8 and 7.5 months in the combination and single-agent groups, respectively (HR for death = 0.61; 95% CI 0.49 to 0.77; *p* < 0.001). Median PFS was 5.6 and 2.4 months, respectively (HR for disease progression or death = 0.44; 95% CI, 0.36 to 0.54; *p* < 0.001). This combination is used as a third-line or later therapy option [115]. By normalizing aberrant, hyperpermeable tumor vasculature, bevacizumab can increase tumor levels of trifluridine, allowing it to impart its DNA-damaging effects. Maintaining persistent VEGF inhibition and suppression of angiogenesis is also beneficial in the setting of metastatic disease [116].

IBI305 is a bevacizumab biosimilar that, in combination with the PD-1 checkpoint inhibitor sintilimab, was approved in China based on the findings of the ORIENT-32 trial. This randomized, open-label, phase II-III trial compared sintilimab–bevacizumab with sorafenib in Chinese patients with HBV-related, unresectable HCC. The sintilimab-bevacizumab biosimilar group had a significantly longer median PFS time of 4.6 months as compared to 2.8 months in the sorafenib group (HR = 0.56; 95% CI 0.46 to 0.7; *p* < 0.0001). This combination also demonstrated an acceptable safety profile [117].

#### 6.1.2. Ramucirumab

Ramucirumab is a monoclonal antibody targeting the VEGFR-2 extracellular domain [118].

The REACH-2 randomized, double-blind phase III trial enrolled patients with HCC and alpha-fetoprotein (AFP) concentrations of 400 ng/mL or greater who had previously received first-line sorafenib. Patients were assigned to receive ramucirumab or a placebo. Median OS was 8.5 months for the ramucirumab group as compared to 7.3 months for the placebo group (HR = 0.71; 95% CI 0.531 to 0.949; *p* = 0.0199). PFS was 2.8 and 1.6 months, respectively (HR for ramucirumab vs. placebo = 0.452; 95% CI 0.399 to 0.603; *p* < 0.0001). Ramucirumab is approved as monotherapy in patients with HCC previously treated with sorafenib [119].

The RAISE trial, a multicenter, randomized, double-blind phase III trial assigned patients with mCRC who had disease progression during or within 6 months of the last dose of first-line therapy to receive either ramucirumab and FOLFIRI or placebo and FOLFIRI. Median OS was 13.3 months in the ramucirumab combination group as compared to 11.7 months in the placebo combination group (HR = 0.844; 95% CI 0.73 to 0.976; log-rank *p* = 0.0219). Survival benefits were consistent across all subgroups in the ramucirumab combination group. Ramucirumab, in combination with second-line FOLFIRI, is approved for the treatment of mCRC after prior treatment with bevacizumab, oxaliplatin, and fluoropyrimidines [120]. The mechanism of synergy is similar to that of other anti-angiogenic monoclonal antibodies, with normalization of aberrant tumor vasculature allowing for better chemotherapy delivery, penetration, and efficacy.

Ramucirumab is used as a second-line treatment for advanced or metastatic gastric and GEJ cancers, either alone or with paclitaxel. JVBD and JVBE were international, double-blind, placebo-controlled studies. JVBD was a monotherapy trial allocating patients to receive ramucirumab or placebo, while JVBE was a combination therapy trial allocating patients to receive ramucirumab or placebo in combination with paclitaxel. OS was increased in patients who received ramucirumab in both the monotherapy [HR = 0.78; 95% CI 0.60 to 0.998; log-rank *p* = 0.047] and combination trials (HR = 0.81; 95% CI 0.68 to 0.96; *p* = 0.017) [121].

The RAINBOW double-blind, randomized phase III trial enrolled patients with advanced gastric or GEJ adenocarcinoma with disease progression on or within 4 months after first-line chemotherapy. Patients were assigned to receive ramucirumab plus paclitaxel or placebo plus paclitaxel. OS was 9.6 months in the ramucirumab combination group as compared to 7.4 months in the placebo combination group (HR = 0.807; CI 0.678 to 0.962; *p* = 0.017 [122]. The RAINBOW trial led to the approval of paclitaxel with ramucirumab as a standard second-line treatment for this patient population [122].

### 6.2. Recombinant Fusion Proteins

#### Ziv-Aflibercept

Ziv-aflibercept is a recombinant fusion protein that blocks VEGF-A, VEGF-B, and PlGF by acting as a decoy receptor. It is approved for patients with mCRC who have progressed on an oxaliplatin-based regimen, used in combination with 5-fluorouracil, leucovorin, and irinotecan (FOLFIRI) chemotherapy [123]. The VELOUR phase III, randomized trial enrolled patients with mCRC previously treated with an oxaliplatin-based regimen. Patients were assigned to receive ziv-aflibercept with FOLFIRI vs. placebo. The median survival time was 13.5 vs. 12.06 months in the ziv-aflibercept and placebo groups, respectively (HR = 0.817; 95% CI 0.713 to 0.937; *p* = 0.0032). Median PFS was 6.9 vs. 4.67 months, respectively (HR = 0.758; 95% CI 0.661 to 0.869; *p* < 0.0001) (*p* < 0.0001) [46,124]. Ziv-aflibercept has since been approved for treatment of this patient population [123].

### 6.3. Tyrosine Kinase Inhibitors

#### 6.3.1. Sorafenib

Sorafenib is an oral multi-kinase inhibitor that targets VEGFR1-3, PDGFR, and RAF kinases [125].

It is approved as a first-line treatment for HCC [126]. In a multicenter, phase III, double-blind trial, patients with advanced HCC who had not received previous systemic treatment were assigned to receive sorafenib or placebo. Median OS was 10.7 months in the sorafenib group as compared to 7.9 months in the placebo group (HR = 0.69; 95% CI 0.55 to 0.87; *p* < 0.001). The median time to radiologic progression was significantly higher in the sorafenib group at 5.5 months as compared to the placebo group at 2.8 months (*p* < 0.001). There was no significant difference in the median time to symptomatic progression at 4.1 and 4.9 months, respectively (*p* = 0.77) [126].

#### 6.3.2. Regorafenib

Regorafenib is an oral multi-kinase inhibitor targeting VEGFR1-3, PDGFR, FGFR, and other kinases involved in tumor growth and angiogenesis [127].

It is approved for use in mCRC patients who have progressed on at least two lines of therapy such as anti-VEGF and anti-EGFR agents [128,129]. The CORRECT trial was an international, multicenter, randomized, placebo-controlled, phase III trial that enrolled patients with mCRC with progression during or within 3 months from last standard therapy. Patients received best supportive care (BSC) along with either regorafenib or placebo. Median OS was 6.4 months in the regorafenib group in comparison to 5 months in the placebo group (HR = 0.77; 95% CI 0.64 to 0.94; *p* = 0.0052) [128]. The CONCUR trial was a randomized, double-blind, placebo-controlled, phase III trial enrolling patients in 25 hospitals in Asia with progressive mCRC who had previously received two or more lines of treatment and assigning them to receive regorafenib or placebo. OS was significantly better in the regorafenib group at 8.8 months as compared to 6.3 months in the placebo group (HR = 0.55; 95% CI 0.4 to 0.77; one-sided *p* = 0.00016) [129].

Regorafenib has been approved for patients with HCC whose tumors have progressed after treatment with sorafenib. The RESORCE trial was a randomized, double-blind, placebo-controlled, phase III trial enrolling patients with HCC who progressed on sorafenib and had Child-Pugh A liver function. Patients were assigned to receive regorafenib or placebo. OS was improved in the regorafenib group at 20.6 months as compared to the placebo group at 7.8 months (HR = 0.63; 95% CI 0.5 to 0.79; one-sided *p* < 0.0001) [130].

The GRID trial was a randomized, double-blind, placebo-controlled phase III study enrolling patients with advanced GISTs refractory to treatment with imatinib and sunitinib. Patients received either regorafenib or placebo. Median PFS per independent blinded central review was 4.8 months in the regorafenib group as compared to 0.9 months in the placebo group (HR = 0.27; 95% CI 0.19 to 0.39; *p* < 0.0001). Regorafenib has been approved as a third-line treatment option in this patient population [131].

#### 6.3.3. Fruquintinib

Fruquintinib is a selective oral VEGFR1-3 inhibitor that is approved for mCRC patients after progression on standard regimens (fluoropyrimidine, oxaliplatin, irinotecan-based chemotherapy, anti-VEGF therapy, and, if medically appropriate, an anti-EGFR therapy). It is typically used in the third-line setting and beyond. The FRESCO-2 international, multicenter, randomized, double-blind, phase III trial enrolled patients with mCRC who had received all current standard therapies and experienced progression on or intolerance of trifluridine-tipiracil or regorafenib. Patients were assigned to receive either fruquintinib or placebo. Median OS was 7.4 months in the fruquintinib group as compared to 4.8 months in the placebo group (HR = 0.27; 95% CI 0.19 to 0.39; *p* < 0.0001) [132].

Furthermore, based on ongoing phase III clinical trials, fruquintinib could be a potential second-line treatment for gastric or GEJ cancer when combined with paclitaxel. The FRUTIGA randomized phase III trial enrolled patients with advanced gastric or GEJ adenocarcinoma who had progressed on fluorouracil and platinum chemotherapy. Patients received either fruquintinib or placebo. Median PFS was 5.6 vs. 2.7 months, respectively (HR = 0.57; 95% CI 0.48 to 0.68; *p* < 0.0001). However, OS was not significantly different at 9.6 and 8.4 months, respectively (HR = 0.96; 95% CI 0.81 to 1.13; *p* = 0.6064) [133].

#### 6.3.4. Sunitinib

Sunitinib, an oral multi-kinase inhibitor, blocks VEGFRs and PDGFRs amongst others [134].

A phase III randomized trial enrolled patients with progressive, well-differentiated pancreatic neuroendocrine tumors (pNETs), with unresectable, locally advanced, or metastatic disease, and assigned them to receive sunitinib or placebo. Median PFS was 10.2 months and 5.4 months, respectively. The objective response rate (ORR) was 9.3% for sunitinib and 0% for placebo. OS data was not obtained as the trial was terminated early due to results favoring sunitinib. It was subsequently approved for treatment in this patient population [135].

The tolerability and efficacy of sunitinib was assessed in a randomized, double-blind, placebo-controlled, multicenter, international trial enrolling patients with advanced GIST who previously experienced resistance to or intolerance of treatment with imatinib. Patients were assigned to receive sunitinib or placebo. A planned interim analysis showing a significantly longer time to tumor progression (TTP) with sunitinib led to early unblinding of the study. Median TTP was 27.3 weeks in the sunitinib group in comparison to 6.4 weeks in the placebo group (HR = 0.33; *p* < 0.0001). Therapy was reasonably well tolerated, and sunitinib was approved as a second-line option for GIST after imatinib failure [131].

#### 6.3.5. Cabozantinib

Cabozantinib is an oral TKI targeting VEGFR-2, RET, and other pathways [136].

A randomized, double-blind, phase III trial enrolled patients with advanced HCC previously treated with sorafenib, who experienced disease progression following at least one systemic treatment. Patients were assigned to receive cabozantinib or placebo. Median OS was 10.2 and 8 months, respectively (HR for death = 0.76; 95% CI, 0.63 to 0.92; *p* = 0.005). Median PFS was 5.2 and 1.9 months, respectively (HR for disease progression or death = 0.44; 95% CI 0.36 to 0.52; *p* < 0.001), and ORRs were 4% and < 1%, respectively (*p* = 0.009). Cabozantinib has been approved as a second-line option for treatment of this patient population [137].

Most recently, the FDA approved cabozantinib for previously treated, unresectable, locally advanced, or metastatic well-differentiated pNETs and well-differentiated extra-pancreatic neuroendocrine tumors (epNETs). A phase III trial enrolled two separate cohorts of patients with pNET and epNET who had previously undergone peptide receptor radionuclide therapy, targeted therapy, or both. Patients were assigned to receive cabozantinib or placebo. In the ePNET cohort, the median PFS with cabozantinib was 8.4 months as compared to 3.9 months with placebo (stratified hazard ratio for progression or death = 0.38; 95% CI 0.25 to 0.59; *p* < 0.001). In the pNET cohort, the median PFS with cabozantinib was 13.8 months, as compared to 4.4 months with placebo (stratified hazard ratio = 0.23; 95% CI 0.12 to 0.42; *p* < 0.001) [138].

#### 6.3.6. Lenvatinib

Lenvatinib is an oral multi-kinase inhibitor that targets VEGFR1-3, RET, FGFR1-4, and PDGFRα [139]. An open-label, phase III, multicenter, non-inferiority trial recruited patients with unresectable HCC, who had not received treatment for advanced disease. Patients were randomly assigned to receive lenvatinib or sorafenib. Non-inferiority was confirmed as a median survival time for lenvatinib of 13.6 months, similar to that of sorafenib at 12.3 months (HR = 0.92; 95% CI 0.79 to 1.06). Lenvatinib was approved in 2018 as a first-line treatment for patients with unresectable HCC [140].

#### 6.3.7. Rivoceranib

Rivoceranib is a small-molecule inhibitor of VEGFR-2 [141]. It is approved in China for use in advanced gastric and GEJ adenocarcinoma as a third-line or later treatment [142]. In the US, rivoceranib has been granted Orphan Drug Designation by the FDA for the treatment of gastric cancer.

The ANGEL international, randomized, placebo-controlled, phase III trial aimed to evaluate the efficacy and safety of rivoceranib and BSC vs. placebo and BSC in previously treated patients with advanced or metastatic gastric or GEJ cancer. OS was not statistically different for rivoceranib vs. placebo at 5.78 and 5.13 months, respectively (HR = 0.93; 95% CI 0.74 to 1.15; *p * =  0.4724). PFS (by blinded independent central review) was improved with rivoceranib at 2.83 months vs. 1.77 months with placebo (HR = 0.58, 95% CI 0.47 to 0.71; *p * <  0.0001). ORRs were 6.5% and 1.3% (*p* = 0.0119), respectively, and disease control rates (DCRs) were 40.3% and 13.2% (*p* < 0.0001), respectively, showing improvement with rivoceranib in both categories [143].

In China, rivoceranib in combination with camrelizumab, an anti-PD-1 antibody, has been approved as a first-line option for advanced HCC. The RESCUE non-randomized, open-label, multicenter, phase II trial evaluated the efficacy and safety of this combination in patients with advanced HCC who were treatment-naïve, refractory, or intolerant to first-line targeted therapy. The ORR was 34.3% (24/70; 95% CI 23.3 to 46.6) in the first-line cohort and 22.5% (27/120; 95% CI 15.4 to 31) in the second-line cohort as assessed by an independent review committee. Median PFS was 5.7 (95% CI 5.4 to 7.4) and 5.5 months (95% CI 3.7 to 5.6), respectively. The 12-month survival rates were 74.7% (95% CI, 62.5 to 83.5) and 68.2% (95% CI, 59.0 to 75.7), respectively [144].

The SPACE phase I study investigated the safety and efficacy of first-line camrelizumab plus rivoceranib and chemotherapy for advanced gastric or GEJ adenocarcinoma. After two phases (dose escalation and dose expansion), the confirmed ORR was 76.5% (95% CI 58.8 to 89.3). Median PFS was 8.4 months (95% CI 5.9 to not evaluable), and median OS was not mature (95% CI 11.6 to not evaluable). This preliminary data showed favorable clinical outcomes and a manageable safety profile in this patient population [145].

**Table 1:** Approved anti-angiogenic agents—monotherapy or primary trials.

Agent	Target	Indication	Trial Outcome	Reference
Ramucirumab	VEGFR-2	2nd line Gastric/GEJ	OS 5.2 vs. 3.8 mo	[146]
Ramucirumab	VEGFR-2	2nd line mCRC	OS 13.3 vs. 11.7 mo	[120]
Ramucirumab	VEGFR-2	2nd line HCC with AFP ≥ 400	OS 8.5 vs. 7.3 mo	[119]
Regorafenib	VEGFR1-3, others	Refractory mCRC	OS 6.4 vs. 5.0 mo	[128]
Regorafenib	VEGFR1-3, others	2nd line HCC	OS 10.6 vs. 7.8 mo	[130]
Lenvatinib	VEGFR1-3, FGFR, others	1st line unresectable HCC	OS 13.6 vs. 12.3 mo (non-inferior to sorafenib)	[147]
Rivoceranib	VEGFR-2	≥3rd line Gastric/GEJ Cancer	OS 5.78 vs. 5.13 mo (NS); PFS 2.83 vs. 1.77 mo; ORR 6.5%; ≥4 L: OS 6.34 vs. 4.73 mo	[143]

**Table 2:** Drug combinations with approved anti-angiogenic agents.

Combination	Agents Involved	Indication	Trial Outcome	Reference
Bevacizumab + FOLFOX4	Bevacizumab, 5-FU, leucovorin, oxaliplatin	2nd line mCRC	OS 12.9 vs. 10.8 mo	[110]
Bevacizumab + atezolizumab	Bevacizumab, atezolizumab	1st line unresectable HCC	OS 19.2 vs. 13.4 mo	[113]
Bevacizumab + trifluridine/tipiracil	Bevacizumab, FTD/TPI	Refractory mCRC	OS 10.8 vs. 7.5 mo	[115]
Ramucirumab + FOLFIRI	Ramucirumab, 5-FU, leucovorin, irinotecan	2nd line mCRC	OS 13.3 vs. 11.7 mo	[120]
Ramucirumab + paclitaxel	Ramucirumab, paclitaxel	2nd line gastric/GEJ cancer	OS 9.6 vs. 7.4 mo	[122]
Camrelizumab + rivoceranib	Camrelizumab, rivoceranib	1st/2nd HCC	ORR 34% (1L), 22% (2L)	[144]
Camrelizumab + rivoceranib + chemo	Camrelizumab, rivoceranib, chemo	1st Gastric/GEJ cancer	ORR 76.5%, PFS 8.4 mo	[145]
Sintilimab + IBI305	Sintilimab, IBI305 (bevacizumab biosimilar)	1st line unresectable HBV-HCC	PFS 4.6 vs. 2.8 mo; OS NR vs. 10.4 mo	[117]

## 7. Emerging Approaches—Targeting the Tumor Microenvironment

There are several emerging therapies that target the TME that should be detailed. These therapies take different shapes and focus on various targets throughout the TME. They include bispecific antibodies, naked antibodies with CD40 agonism effects or TGF-β targeting, cellular therapies such as chimeric antigen receptor (CAR)-Ts, and next-generation TKIs.

The emerging approaches that are detailed below are all attempting to address the challenges in developing treatments that successfully modulate the TME of GI cancers. While some angiogenesis inhibitors and kinase inhibitors such as bevacuzimab and lenvatinib have demonstrated some success in combination with ICIs, these combinations have not been universally efficacious. One possibility is that diverse TME cells and targets exist, leading to resistance against lenvatinib and necessitating new treatments that can overcome this persistent suppressive TME. For example, Tregs and MDSCs are driven by receptors and pathways beyond VEGFR, RET, PDGFR, and FGFR, therefore necessitating new molecules that can successfully target alternate pathways. Other therapeutic strategies to overcome negative TMEs may be to combine complementary targeting mechanisms into single molecules.

### 7.1. Kinase Inhibitor Combinations

Based on the success of combining kinase inhibitors and ICIs in multiple solid tumors such as HCC, this treatment strategy has been tested extensively in other GI cancers such as CRC and pancreatic ductal adenocarcinoma (PDAC). However, the majority of these trials failed to show benefits over active comparators. The generally cold TME and multiple mechanisms of immune suppression likely play significant roles in preventing immunomodulation by most kinase inhibitors.

#### Zanzalintinib with Atezolizumab

Zanzalintinib, formerly known as XL092, is a novel TKI with activity against several oncogenic kinases, including VEGFR2, MET, TYR, AXL, and MER. Suppression of these targets causes tumor growth inhibition, decreased angiogenesis, and increased T-cell infiltration in murine models of CRC [148]. These findings coincided with decreased MDSCs, Tregs, and repolarization of TAMs, suggesting that the broad inhibitory activity of zanzalintinib is indeed causing beneficial immunomodulation of the TME.

Currently, zanzalintinib is being studied in combination with the anti-PD-L1 atezolizumab for the treatment of mCRC. The ongoing STELLAR 303 trial (NCT05425940) is an international open-label, randomized clinical trial that assigned patients with mCRC who had progressed on standard of care chemotherapy to receive zanzalintinib plus atezolizumab vs. regorafenib, the active control arm. Critically, this study was conducted on patients with MSS mCRC, a highly immune cold tumor [149]. The study has fully accrued and is now undergoing survival analysis. Due to the overall lack of success in ICI combinations for MSS CRC, the results are eagerly anticipated.

### 7.2. Bispecific Antibodies

Bispecific antibodies (bsAbs) are a promising development, with their ability to target two different antigens allowing for cooperative binding to impart a greater therapeutic effect than traditional monoclonal antibodies (mAbs). In the context of the complex and multifaceted landscape of the TME, bsAbs offer the advantage of modulating multiple components at one time. Unlike mAbs, which bind only a single antigen, bsAbs can redirect immune effector cells, such as T-cells or NK cells, to tumor cells while concurrently blocking immunosuppressive signals within the TME [150]. This dual functionality enables a more potent anti-tumor effect, improved immune cell infiltration, and potential synergy with existing immunotherapies. When targeting a signaling pathway, bsAbs hold the advantage over mAbs by being able to bind to two targets within the same pathway, potentiating downstream inhibition, and potentially overcoming resistance [151]. BsAbs represent a particularly useful strategy in GI malignancies, as they frequently do not benefit significantly from conventional mAbs [152,153].

#### 7.2.1. Ivonescimab

Ivonescimab is a tetrameric bsAb targeting PD-1 and VEGF. A phase Ia dose escalation study showed manageable safety profiles with the most common grade ≥3 treatment-related adverse events being hypertension, increased alanine aminotransferase and aspartate aminotransferase, and colitis. Promising efficacy signals were observed in multiple solid tumors, including mismatch repair-proficient (pMMR) ones. Of the nine patients with pMMR CRC treated with 3 mg/kg of the drug, one achieved a partial response and two achieved stable disease for 8 and 16 weeks, respectively. The patient with partial response was heavily pre-treated and bevacizumab-experienced [154].

More recently, an open-label, multicenter, randomized phase II trial was initiated, assigning patients with mCRC to receive a combination of FOLFOXIRI + ivonescimab with and without ligufalimab, a novel humanized IgG4 mAb targeting CD47. Preliminary data has demonstrated promising efficacy and tolerable safety, with an ORR of 81.8% in the group without ligufalimab and 88.2% in the group with ligufalimab [Deng]. While the median PFS and OS rates were not yet mature, the PFS at 9 months was found to be 81.4% (95% CI 52.1 to 93.7) and 86.2% (95% CI 55 to 96.4) in the groups without and with ligufalimab, respectively [155]. The early-stage data from the aforementioned phase I and II trials suggest promising activity of PD-1 and VEGF bsABs in CRC.

Currently, there are two phase II clinical trials involving ivonescimab efficacy in the setting of GI cancers still in the recruitment phase [151]. Clinical trial NCT06848842 will evaluate the efficacy of ivonescimab with chemotherapy in unresectable mCRC with liver metastases. Clinical trial NCT06375486 will evaluate the efficacy of ivonescimab with hepatic artery infusion chemotherapy in unresectable HCC.

#### 7.2.2. Cadonilimab

Cadonilimab is one of the novel bsAbs currently being studied for use in GI malignancies. It is a symmetric, tetravalent bsAb with a crystallizable fragment (Fc)-null design, and it targets both CTLA-4 and PD-1. Its structure prevents binding to Fcγ receptors and complement proteins, resulting in the silencing of antibody-dependent cellular cytotoxicity and complement-dependent cytotoxicity, which can ultimately result in reduced systemic inflammation and decreased numbers of immune-related adverse events [156,157]. By acting to inhibit both immune checkpoints, cadonilimab aims to improve antitumor immunity via direct TME modulation [156].

Its safety and objective response rate were assessed in the COMPASSION-03 phase Ib and phase II trial conducted in China on patients with various forms of GI malignancies including HCC and esophageal squamous cell carcinoma. This study showed a promising tumor response rate as well as a reasonable safety profile [158]. From here, a phase III trial studied patients with advanced gastric or GEJ adenocarcinoma and found that treating patients with cadonilimab improved survival time [159]. This blockade of dual inhibitory pathways results in improved survival and proliferation of CD8+ and CD4+ T-cells, allowing for their ability to continue their effector functions within the cancer. It also results in an increased ratio of effector T-cells vs. Tregs and MDSCs, leading to increased inflammation within the TME [160]. The development of this agent continues in more advanced trials.

#### 7.2.3. KN026/KN046 Combination

Two bsAbs are being studied together in HER2-positive GI malignancies. KN026 is an anti-HER2 bsAb while KN046 is a dual PD1/CTLA-4 ICI. Two studies (NCT04040699 and NCT04521179) involving 38 patients with HER2-positive, microsatellite stable (MSS) gastric orGEJ cancer received therapy with KN026 and KN046. The study exhibited an ORR of 78.9% (95% CI 62.7 to 90.4) with one complete response and twenty-nine partial responses. The median PFS was 11 months (95% CI 5.5 to 16.5). HER2-positive tumors have been shown to have reduced frequencies of exhausted and dysfunctional CD8+ T-cells as compared to HER2-negative tumors, suggesting that this antibody combination can be especially beneficial in enhancing antitumor response in HER2-positive tumors. This study also found that this treatment led to increases in the presence of intra-tumoral DCs, NK cells, and effector T-cells in addition to a decrease in Treg populations, showing that its TME modulating effects have a beneficial anti-tumor profile [161].

### 7.3. CD40 Agnonism

CD40 is a cell-surface protein included within the TNF receptor family and is expressed on APCs such as dendritic cells and macrophages. Antibodies that target and subsequently activate CD40 can result in enhanced DC maturation and thus improved antigen presentation, activation, and proliferation of CD8 T-cells, a shift from the macrophage M2 (immunosuppressive) phenotype to the M1 (pro-inflammatory) phenotype, and an increase in the production of inflammatory cytokines, such as CXCL10 and CCL22 [162,163,164].

The following three CD40 agonist antibodies each act to modulate the TME to improve anti-tumor immunity, despite differences in their designs and effects. Both mitazalimab and sotigalimab are FcγR-dependent while selicrelumab is FcγR-independent. Mitazalimab acts on dendritic cells and B-cells and expands CD8+ T-cells, while sotigalimab exerts effects on Treg population depletion and the expansion of tumor-specific T-cell clonotypes [165,166,167,168]. With selicrelumab, the result is a strong intratumor T-cell infiltration, but its dosing needs to be significantly limited because of the risk of cytokine release.

#### 7.3.1. Selicrelumab

Selicrelumab is a mAb that targets CD40. Preclinical studies showed that this antibody was well tolerated when given to M. fascicularis monkeys in both subcutaneous and intravenous forms. These studies demonstrated evidence of increased circulating T- and B-cells in both the blood and bone marrow with a concurrent reduction in macrophages, indicating that this CD40 agonist is capable of instigating a systemic immune response [169]. This antibody was subsequently studied in human patients. One such study involved 16 patients with resectable PDAC alongside the standard chemotherapy regimen. Following exposure, the tumor site was found to have large numbers of T-cells as well as increases in DC number and M1 macrophages and the depletion of tumor stroma, all of which are associated with T-cell activation and their peripheral expansion. This study showed that treatment with selicrelumab resulted in significant beneficial changes to the TME [170]. Another phase I study focusing on PDAC demonstrated that selicrelumab in conjunction with gemcitabine not only was well tolerated but also presented anti-tumor activity. There was an increased systemic immune response that included leukocyte trafficking, production of cytokines, and cellular activation, all of which support this antibody serving as a potent modulator of the TME of GI malignancies [171].

#### 7.3.2. Sotigalimab

Another notable CD40 agonist includes sotigalimab. In preclinical studies, it was found to work synergistically with chemotherapy to have a significant macrophage-dependent effect on the TME, particularly the stroma, and on tumor regression, without needing to utilize the effect of T- or B-cells [172]. Based on this rationale, a phase Ib trial assessed the utility of sotigalimab administration following a standard regimen of gemcitabine and nab-paclitaxel in untreated metastatic pancreatic adenocarcinoma. Patients received increasing doses of sotigalimab (0.1 mg/kg, 0.3 mg/kg), with some cohorts also receiving nivolumab 240 mg IV. This combination was found to have a tolerable safety profile, including two febrile neutropenia dose-limiting toxicities (DLTs). Within the 23 DLT-evaluable patients, the ORR was 58%, and PBMC profiling revealed that there was modulation of both dendrites and B-cells in addition to CD4+ and CD8+ T-cell activation. Though this study was limited in its design, due to small cohort size, the absence of randomized controls, and possible selection bias, it highlights the encouraging role that CD40 agonism plays in potential treatment of metastatic pancreatic cancer.

#### 7.3.3. Mitazalimab

Mitazalimab is another CD40 agonist studied for its role in activating APCs and enhancing the anti-tumor immune response. In preclinical studies, mitazalimab has been shown to induce activation of DCs and lead to more effective T-cell-mediated immunity in murine models, resulting in improved tumor control. The phase Ib/II OPTIMIZE-1 trial studied mitazalimab in conjunction with a modified FOLFIRINOX regimen in patients with untreated PDAC. At doses of 900 µg/kg, mitazalimab combined with chemotherapy was found to have a manageable safety profile, without any DLTs. The ORR was approximately 40%. Median OS was 14.9 months, with a confirmed duration of response of 12.6 months and an 18-month OS rate of 36.2% [173]. A registrational phase III trial is planned. The aforementioned data shows that mitazalimab can potentially serve to play a role in the conversion of tumors traditionally seen as “cold” into immunogenic targets.

### 7.4. Cellular Therapies

Though novel cellular therapies pose an exciting prospect, it must be noted that the manufacturing process can prove challenging in their clinical use. Methods such as chimeric antigen receptor T-cell (CAR-T) therapy rely on autologous starting materials collected from cancer patients. Factors such as age, disease pathology, and prior treatment can affect both the quality and quantity of starting materials [174]. After collection and isolation, T-cells must be expanded ex vivo. The presence of other cells, such as monocytes, granulocytes, and MDSCs, at culture initiation can interfere with the activation, proliferation, and expansion of T-cells [175]. Despite these challenges, much work is going into optimizing cellular therapies for widespread clinical use as they represent another step forward in precision medicine.

#### 7.4.1. Satricabtagene Autoleucel

Satricabtagene autoleucel is a claudin 18.2-targeting cellular therapy being studied in GI malignancies. A phase I trial evaluated its efficacy in advanced claudin-expressing gastric, GEJ, and pancreatic cancers. Eighty-three participants were enrolled. The drug showed consistent efficacy in addition to a tolerable safety profile, with an ORR of 38.8% and a disease control rate of 91.8%. Median PFS was 4.4 months, and OS was 8.8 months. This therapy showed reasonable therapeutic potential without concern for overt harm towards patients with advanced GI cancers [176]. A phase II trial in China studied a similar population with advanced gastric or GEJ patients refractory to more than two previous lines of treatment. In the intention-to-treat population, median PFS was 3.25 months in the satricabtagene autoleucel group and 1.77 months in the control chemotherapy group (HR = 0.37; 95% CI 0.24 to 0.56; one-sided log-rank *p* < 0.0001) [177].

#### 7.4.2. Mesothelin CAR-T

Mesothelin-specific CAR-T targets the cell surface antigen mesothelin, which is overexpressed in many solid tumors, with limited expression in non-tumor tissue [178]. This mode of therapy has been studied in PDAC. In a small study, six patients with chemo-refractory metastatic PDAC received this therapy via regional infusion into the peritoneal cavity. The treatment was well tolerated by the patients and did not have any dose-limiting toxicities. The study also found evidence of CAR-T cell expansion into peripheral blood, and several patients were found to have evidence of stable disease for several months. While there are still limited clinical trials of this therapy, there are several small clinical and preclinical studies that have shown improved targeting of both the tumor and surrounding stroma, specifically within these PDAC models, indicating that this therapy would be useful for its TME-modulating effects [178,179]. Wehrli et al. demonstrated that mesothelin-specific CAR-T cells outperformed control CAR-T cells when used in co-culture with PDAC and CAFs [178].

#### 7.4.3. GUCY2C-Targeted CAR-T

GUCY2C-targeted CAR-T therapy is another emerging cellular therapy being studied that targets a surface antigen overexpressed in CRC on the luminal surface of intestinal mucosa [180]. A small study focused on nine patients with mCRC who were given this treatment following lymphodepletion. It found no dose-limiting toxicities or neurotoxicity and only grade 1-2 cytokine release syndrome in three patients. Five patients achieved stable disease. Of these five patients, one notably had prolonged disease control. The CAR-T cells were found to persist for up to 56 days [181]. There are some notable preclinical studies as well, including a study using a murine model of CRC. This study found that humanized GUCY2C CAR-T cells reduced tumor burden and improved survival in mice with GUCY2C-expressing mCRC. Furthermore, therapeutic effects were achieved without causing immune-mediated damage to surrounding intestinal tissues or extraintestinal tissues, supporting the safety and effectiveness of this treatment [180].

#### 7.4.4. Other Cellular Therapies

Additional personalized therapies, including T-cell receptor (TCR)-T and tumor-infiltrating lymphocyte (TIL) therapies, are currently being studied for their activity in GI cancers. A phase II trial of a personalized neoantigen TCR-T cell therapy that targets patient-specific CRC mutations found that 3 out of the 7 patients studied had objective regressions, which confirms the feasibility and utility of this treatment [182]. Another type of therapy currently studied is gavocabtagene autoleucel, which is a mesothelin-directed T-cell receptor fusion construct, which was found to generate a 23% partial response rate in tumors containing high levels of mesothelin, including GI malignancies [183]. From a safety perspective, this therapy also resulted in cytokine release syndrome and pneumonitis in a dose-dependent fashion, necessitating further data to better assess its safety profile [183]. TCR-T therapies that target multiple antigens, such as the shared-target MAGe-A4/PRAME, have been found to provide disease stabilization in GI patients with a tolerable side effect profile as well [184]. Finally, these therapies include TIL therapy. A recent study focused on patients treated with TILs plus pembrolizumab. This treatment resulted in an ORR of 7.7–23.5% in a pMMR GI cancer cohort [185]. These studies are still early in their design and not without limitations. For each treatment, patient-specific neoantigens must be identified and then the high affinity TCR cells must be developed in addition to determining ways to overcome the immunosuppressive TME that has been discussed above as well as the cytotoxicity of these treatments.

### 7.5. TGF-β Targeting

TGF-β promotes EMT, a process where epithelial cells transform into mesenchymal cells, acquiring migratory and invasive properties. It also stimulates the excessive deposition of ECM components, leading to tissue stiffening and scar formation. Furthermore, TGF-β induces the formation of CAFs, which are critical in supporting tumor growth and metastasis. Together, these effects contribute significantly to the progression of fibrosis and various cancers [186].

#### 7.5.1. SAR439459

Targeting TGF-β has been challenging and disappointing so far. SAR439459 is a second-generation mAb that targets all TGF-β isoforms. An expansion phase Ib trial included HCC and CRC patients. The response rate was low (8% in the large cohort) with no significant association between clinical response and plasma TGF-β levels at baseline. In total, 11 out of 14 patients with HCC (79%) had a hemorrhagic adverse event. The study was discontinued due to a lack of efficacy and high bleeding risk [187].

#### 7.5.2. Bintrafusp Alfa

Bintrafusp alfa is a bifunctional fusion protein composed of a mAb against PD-L1 fused to a TGF-β trap. A randomized phase II/III trial compared the safety and efficacy of bintrafusp alfa in combination with gemcitabine + cisplatin to gemcitabine + cisplatin alone in patients with biliary tract cancer. The trial included 297 patients and showed a comparable median OS between the bintrafusp alfa group (11.5 months) and the placebo group (11.5 months). The most common severe adverse event related to treatment was anemia, and bleeding events were more frequent in the bintrafusp alfa group [188].

Heightened bleeding risk and poor responses are hurdles that need to be overcome for TGF-β targeting to advance in development. Despite these obstacles, TGF-β targeting remains a viable potential route for combatting disease and continues to be studied due to its significant role in cancer growth and development.

## 8. Emerging-Approaches and Potential Angiogenic Inhibitors

Similarly to emerging TME modulators (see Section 7), prior anti-angiogenic therapies suffer from a limited understanding of the complex biology of tumor angiogenesis. Due to the presence of targets beyond VEGF and VEGFR1-3, novel agents are being developed that inhibit angiogenesis through diverse targets and pathways. BsAbs are playing a major role in this field, as these types of antibodies allow for convenient targeting of different angiogenesis-based molecules using a single drug. An important remaining challenge will be to determine biomarkers of activity that can aid in distinguishing responders from non-responders. Newer analytical techniques such as spatial omics, digital pathology, and single-cell omics may aid in unraveling the complex biological interplay between angiogenesis and the TME, thereby yielding a new generation of biomarkers for these drugs and combinations.

Table 3 lists the numerous TME-directed and anti-angiogenic agents reviewed in Section 7 and Section 8 that are currently evolving in the pipeline.

### 8.1. The DLL4/Notch Signaling Pathway

Targeting the DLL4/Notch pathway may offer a new strategy for inhibiting angiogenesis, as it works through a mechanism different from VEGF inhibitors [189]. Blocking this pathway promotes abnormal, non-productive blood vessel formation in tumors. Although vascular density increases, blood flow is poor, oxygen levels drop, and tumor growth slows. Importantly, this effect has also been seen in tumors that progressed on anti-VEGF therapy [189,190]. There are currently multiple bsAbs targeting the DLL4/Notch pathway in development.

#### 8.1.1. Tovecimig

Tovecimig is a bsAb built on a VEGF-targeting backbone, like bevacizumab, linked to a fragment that binds DLL4 [191]. This drug showed greater anti-tumor effects in several human cancer models compared to targeting VEGF or DLL4 alone [191,192]. A study by Kim, D. et al. confirmed its effectiveness in preclinical models of advanced gastric cancer, finding that this bsAb had significant effects on tumor growth, namely inhibiting cancer stem cell replication [193]. This antibody was found to be effective in both xenograft and orthotopic mouse models, leading to further study in phased trials. Ongoing research is exploring tovecimig in GI cancers. For instance, a phase II trial combining tovecimig with paclitaxel in Korean patients with biliary tract cancers reported an encouraging ORR of 37.5% (95% C: 18.8 to 59.4) [194]. COMPANION-002 is an ongoing registrational, open-label, randomized phase II/III trial enrolling patients with pre-treated biliary tract cancers in the United States and treating them with paclitaxel and tovecimig vs. paclitaxel monotherapy [195].

#### 8.1.2. Navicixizumab

Navicixizumab is a bsAb that blocks both VEGF and DLL4 [196]. In a phase Ia study involving patients with solid tumors who had received multiple prior treatments, including bevacizumab, navicixizumab was well tolerated and showed early signs of anti-cancer activity [196]. Although the follow-up phase 1b study was stopped early, results from combining navicixizumab with FOLFIRI in second-line treatment of mCRC showed promising disease control [197]. After this, research shifted more toward ovarian cancer [198]. In platinum-resistant ovarian cancer, navicixizumab combined with paclitaxel showed clinical benefits in both patients who had and had not previously received bevacizumab [198].

### 8.2. The Angiopoietin-2/Tie Pathway

Another mechanism of tumor resistance involves the upregulation of angiopoietin-2 (Ang-2). Preclinical studies have shown that tumors can bypass natural angiogenesis inhibitors by increasing Ang-2 levels [199]. In fact, treatment with anti-VEGF-A agents has also been shown to cause a rise in Ang-2 expression, both in laboratory and clinical models [200,201]. Blocking Ang-2 in preclinical studies reduced the number of blood vessels in tumors and suppressed tumor growth [54,202,203]. Drugs targeting the Ang-2 pathway function through a variety of mechanisms.

#### Vanucizumab

Vanucizumab is a bsAb that targets VEGF-A (using a bevacizumab-derived arm) and Ang-2 (using an LC06-derived arm) [204]. Preclinical studies demonstrated strong antiangiogenic, antitumor, and antimetastatic effects, prompting a phase I trial in patients with advanced solid tumors, including GI cancers (NCT01688206) [204]. The drug showed a favorable safety profile and encouraging biological activity [205]. However, in the McCAVE trial comparing vanucizumab plus mFOLFOX-6 to bevacizumab plus mFOLFOX-6 in untreated mCRC, there was no improvement in PFS. Additionally, vanucizumab led to more GI side effects [206]. These findings suggest that more research is needed to fully understand the potential efficacy of vanucizumab across different GI tumor types [205].

### 8.3. Hypoxia-Inducible Factors

HIFs help control how cells respond to low oxygen levels. They include an oxygen-sensitive part (2α) and another part (1β) that is a constitutively expressed subunit. When oxygen is low, HIF-2α links with HIF-1β to form a complex that triggers the activity of genes involved in red blood cell production, new blood vessel formation, and cell growth, which can help tumors develop [207]. HIFs can activate many genes that promote blood vessel growth, such as VEGF, its receptors FLT-1 and FLK-1, PDGF-B, PAI-1, TIE-2, MMP-2, MMP-9, and angiopoietins like ANG-1 and ANG-2 [208]. High levels of HIF-2α have been linked to worse outcomes and faster tumor growth in cancers such as CRC and HCC [209,210,211].

Belzutifan is a pill that blocks HIF-2α [207]. It is approved to treat advanced kidney cancer after treatment with PD-1 or PD-L1 blockers and VEGF-targeting drugs [212]. More recently, it was approved by the FDA for patients with locally advanced, unresectable, or metastatic pheochromocytoma or paraganglioma [213]. Ongoing research is exploring the potential use of Belzutifan in treating GISTs and pNETs (NCT04924075) [214].

**Table 3:** Emerging therapies.

Agent	Mechanism of Action	Target	Clinical Stage	Indication	References
Zanzalintinib + atezolizumab	Tyrosine kinase inhibitor + anti-PD-L1	TME and angiogenesis	Phase III	mCRC	[149]
Ivonescimab	Bispecific antibody targeting PD-1 and VEGF	TME and angiogenesis	Phase I/II	Unresectable CRC, unresectable HCC	[151,155]
Cadonilimab	Bispecific antibody-targeting PD-1 and CTLA-4	TME	Phase I/II/III (COMPASSION-03, gastric and esophageal cancer)	Esophageal SCC, gastric/GEJ adenocarcinoma	[159]
KN026/KN046	Bispecific antibodies: HER2 + PD-1/CTLA-4	TME	Phase II	HER2+ gastric/GEJ, HER2+ CRC	[161]
Selicrelumab	CD40 agonist antibody	TME	Phase I in resectable and metastatic PDAC	Pancreatic cancer	[170]
Sotigalimab	CD40 agonist antibody (FcγR-dependent)	TME	Phase Ib (gem+nab-paclitaxel ± nivolumab)	Pancreatic cancer	[172]
Mitazalimab	CD40 agonist antibody	TME	Phase Ib/2 (OPTIMIZE-1), Phase I	Pancreatic cancer	[165]
Satricabtagene autoleucel	CAR-T-targeting Claudin 18.2	TME	Phase I/II	Gastric, GEJ, pancreatic cancer	[176]
Mesothelin-specific CAR-T	CAR-T-targeting mesothelin	TME	Small study, early clinical	Pancreatic cancer	[178]
GUCY2C CAR-T	CAR-T-targeting GUCY2C	TME	Early phase	CRC	[181]
SAR439459	TGF-β antibody	TME	Phase Ib (discontinued)	HCC, CRC	[187]
Bintrafusp alfa	Bifunctional fusion protein-,targeting PD-L1 and TGFβ	TME	Phase II/III	Biliary tract cancer	[188]
Tovecimig	Bispecific antibody-targeting VEGF and DLL4	TME and angiogenesis	Phase II with paclitaxel in BTC (COMPANION-002); Phase Ib/IIa planned with chemo/PD-1	Gastric, biliary tract cancer	[192]
Navicixizumab	Bispecific antibody blocking VEGF and DLL4	TME and angiogenesis	Phase Ia/Ib; development shifted toward ovarian cancer	CRC	[196]
Vanucizumab	Bispecific antibody-targeting Ang-2 and VEGF-A	TME and angiogenesis	Phase I and McCAVE Phase II/III in mCRC	CRC	[206]
Belzutifan	HIF-2α inhibitor	TME	FDA approved (RCC, paraganglioma); trials in GIST and pNETs	GIST, pNET	[215]

## 9. Conclusions

GI malignancies represent a significant global health burden, with pathogenesis driven largely by aberrant angiogenesis and a dynamic TME. Advances in our understanding of these biological processes have facilitated the development of targeted therapies designed to disrupt the supportive network that tumors exploit for metastasis and survival. Current therapies, including anti-VEGF agents, TKIs, and ICIs, have shown clinical benefit in select GI cancer populations. Despite progress, limitations remain in the availability of effective treatment options for many patients. Specifically, the limited success of immunotherapies in MSS tumors highlights a need for strategies that allow the TME to be more permissive to immune infiltration and response. Additionally, the reliance on cytotoxic chemotherapy combinations in advanced stage disease often leads to cumulative toxicities, compromising patient quality of life and treatment adherence. Future solutions to this issue can be found through the development of combination regimens that integrate chemotherapy with immunotherapy or anti-angiogenic agents in a way that minimizes overlapping toxicities. This can be performed through sequential administration or the use of less toxic chemotherapeutic backbones [216].

Emerging strategies are increasingly focusing on dual-targeting bispecific antibodies, CD40 agonism, cellular therapies such as CAR-T and TILs, and novel angiogenesis inhibitors beyond VEGF, such as those targeting DLL4/Notch and angioprotein-2 pathways. These approaches aim to not only directly inhibit tumor growth but also reprogram the TME into a more immunologically active state, thereby enhancing anti-tumor responses. Early-phase clinical trials have shown promising results, suggesting that a multi-modal approach that integrates immunomodulation and anti-angiogenesis could redefine treatments for GI cancers. Future directions should include expanded biomarker development to enable better patient stratification and prediction of response, along with more integrative translational research to avoid mechanisms of resistance and immune evasion. Advancement in personalized medicine such as molecular profiling and real-time monitoring will be important factors in optimizing therapy selection and timing [217]. A concerted effort in both clinical and translational research will be critical to transform preclinical innovation into durable and widely accessible clinical treatment options for patients with GI malignancies.

## Data Availability

Not applicable.

## Supplementary Materials

The following supporting information can be downloaded at: https://www.mdpi.com/article/10.3390/ph18081160/s1, Table S1: Drug Chemical Structures.
