# Supplementary material for: Emerging Strategies for Targeting Angiogenesis and the Tumor Microenvironment in Gastrointestinal Malignancies: A Comprehensive Review
Source: Pharmaceuticals (Basel). 2025 Aug 5;18(8):1160. doi: 10.3390/ph18081160 (PMC12389621; doi:10.3390/ph18081160)
Supplement: Supplementary file 1 [file pharmaceuticals-18-01160-s001.zip › pharmaceuticals-3735712-supplementary.pdf]

Supplemental Table S1: Drug Chemical Structures.

Drug	CAS Registry Number	Molecular Formula	Structure
Oxaliplatin	61825-94-3	$C_8H_{12}N_2O_4Pt$	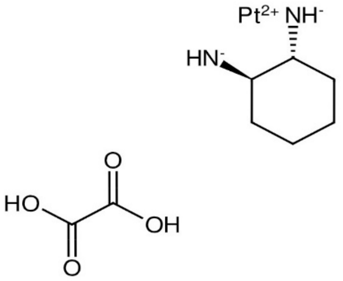
5-fluorouracil	51-21-8	$C_4H_3FN_2O_2$	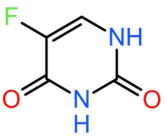
Paclitaxel	33069-62-4	$C_{47}H_{51}NO_{14}$	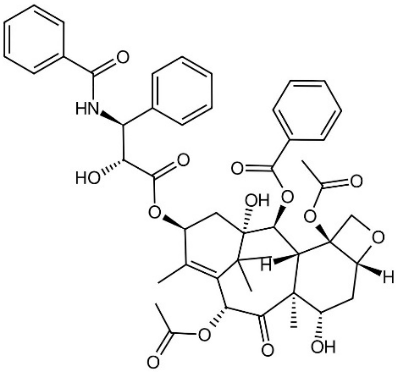
Sorafenib	284461-73-0	$C_{21}H_{16}ClF_3N_4O_3$	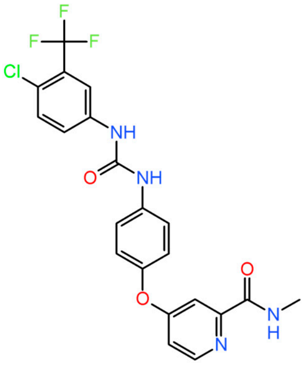

Regorafenib	755037-03-7	$C_{21}H_{15}ClF_4N_4O_3$	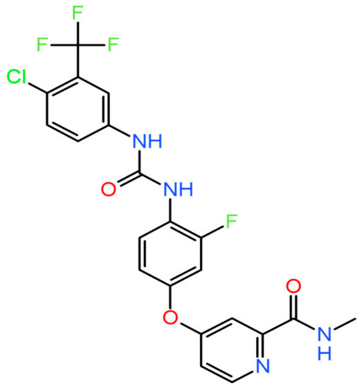
Fruquintinib	1194506-26-7	$C_{21}H_{19}N_3O_5$	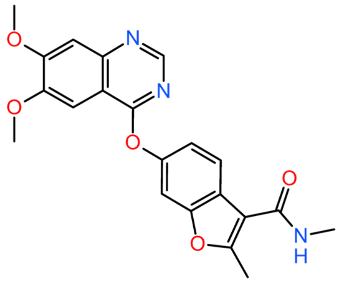
Sunitinib	557795-19-4	$C_{22}H_{27}FN_4O_2$	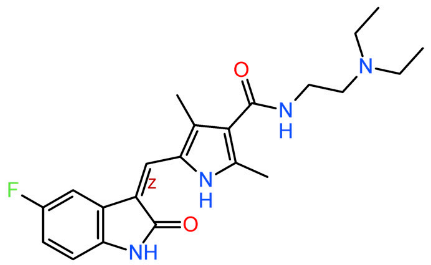
Cabozantinib	849217-68-1	$C_{28}H_{24}FN_3O_5$	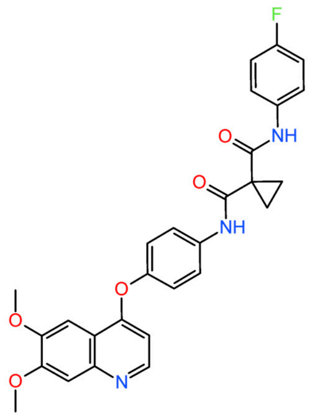

Lenvatinib	417716-92-8	$C_{21}H_{19}ClN_4O_4$	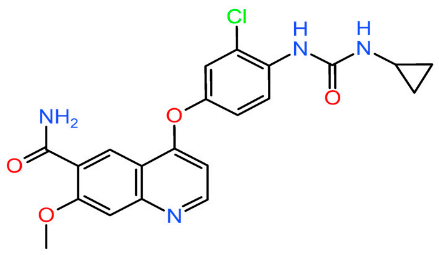
Rivoceranib	811803-05-1	$C_{24}H_{23}N_5O$	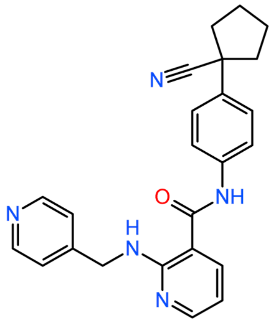
Irinotecan	97682-44-5	$C_{33}H_{38}N_4O_6$	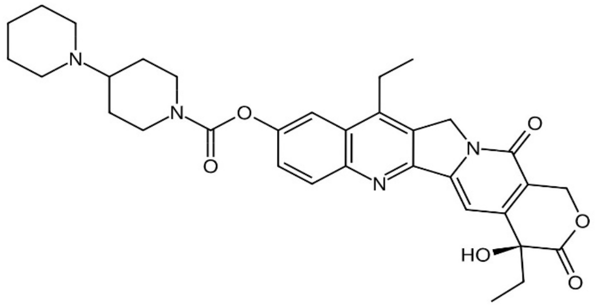
Belzutifan	1672668-24-4	$C_{17}H_{12}F_3NO_4S$	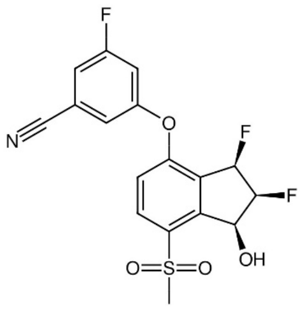
Molecular formulas and chemical structures were retrieved from PubChem and DrugBank			
